# When to remove implantable vascular access ports? a retrospective analysis of 376 patients with breast cancer and implantable vascular access ports

**DOI:** 10.1186/s12893-025-03173-4

**Published:** 2025-10-03

**Authors:** Jun Luo, Zheng Yao, Weiren Liang, Gu Zhao, Jiaping Zheng, Yuwei Liu, Zifang Jiang, Xinyan Yu

**Affiliations:** 1 Postgraduate training base, Alliance of Wenzhou Medical University, (Zhejiang Cancer Hospital), No.1 East Banshan Road, Gongshu District, Hangzhou, Zhejiang 310022 China; 2 Zhejiang Cancer Hospital, Hangzhou, Zhejiang 310022 China; 3 Hangzhou Institute of Medicine (HIM), Chinese Academy of Sciences, Hangzhou, Zhejiang 310022 China

**Keywords:** Port catheters, Breast cancer, Device removal, Complications, Retrospective study

## Abstract

**Background:**

The optimal timing of removal of implanted vascular access ports (PORTs) in cancer patients is unknown. This study aimed to explore the timing of PORT removal and the impact of PORT removal within 2 years in breast cancer patients.

**Methods:**

This retrospective study included breast cancer patients who underwent PORT implantation at our Hospital between July 2012 and May 2022 and ultimately had their PORTs removed. QoL scores were assessed using the Karnofsky method. The primary endpoint was quality of life (QoL) (A: 80–100 points, basically normal self-care ability; B: 60–79 points, mild dependence on self-care ability; C: <60 points, moderate to severe dependence). The secondary endpoints included the proportion of unplanned PORT removal, secondary catheterization, medical costs, and complications.

**Results:**

This study included 376 female patients with a median age of 52 (range, 22–72) years. The median PORT indwelling time was 464 (range, 16-2717) days. No significant differences were observed in the QoL scores between patients with PORT indwelling time ≤ 2 vs. >2 years [A/B/C: 90.87%/7.94%/1.19% vs. 95.97%/4.03%/0, *P* = 0.185]. Patients with PORT indwelling time ≤ 2 years exhibited higher probabilities of unplanned port removal (16.76% vs. 3.99%, odds ratio = 2.42, *P* = 0.004), higher secondary catheterization (28.99% vs. 3.72%, odds ratio = 5.08, *P* = 0.004), and higher medical costs (24.57 ± 31.36 vs. 7.16 ± 1.19 CNY/day, *P* < 0.001) compared with patients with PORT indwelling time > 2 years. There were no significant differences in the frequency of complications between groups (*P* = 0.751). In the subgroup of patients with planned PORT removal, the ≤ 2-year group still showed significantly higher secondary catheterization rates (31.22% vs. 8.26%, *P* < 0.001) and daily costs (14.83 vs. 6.83 CNY/day, *P* < 0.001).

**Conclusion:**

In patients with breast cancer who do not require regular intravenous administration, it may be recommended to consider removing the infusion port after 2 years to reduce the risks of secondary catheterization and reduce medical costs, unless irreversible adverse events affect the correct use of the PORT.

**Clinical trial number:**

Not applicable.

**Supplementary Information:**

The online version contains supplementary material available at 10.1186/s12893-025-03173-4.

## Background

Breast cancer is the most prevalent malignancy among women, with an estimated 2,261,419 new cases in 2020 worldwide [1, 2]. Breast cancer management is multidisciplinary and includes surgery, chemotherapy, hormonal therapy, targeted therapy, immunotherapy, and radiotherapy [3–6]. Intravenous drugs (i.e., chemotherapy, some targeted therapies, and immunotherapy) remain crucial therapeutic approaches for patients with breast cancer [3–6]. Still, venous access can be difficult in some patients because of small, mobile, or fragile veins, and age and previous chemotherapy can exacerbate the problem. Chemotherapy extravasation is a serious complication of intravenous treatments and can result in local necrosis and the need for resection and suboptimal systemic drug concentrations [7]. Centrally inserted totally implanted vascular access ports (PORTs) have gained recognition as long-term implantable devices offering a completely internal venous access pathway, known as totally implantable venous access devices (TIVAD) [8]. With technological advances and evolving perspectives, the use of PORTs has become increasingly widespread in recent years. Nevertheless, it is essential to acknowledge that PORTs are implanted devices within the human body. Prolonged PORT retention inevitably raises the risks of catheter displacement, breakage, infections, and thrombosis [9–11].

For patients who have completed their therapy, PORT removal can enhance their quality of life [12], but the optimal timing for PORT removal remains a crucial question. PORT removal involves a surgical procedure with varying difficulty levels and associated risks for different patients. The physicians must comprehensively consider the risk-benefit ratio of PORT removal before deciding. Currently, research on the timing of PORT removal remains relatively scarce [13, 14]. Given the high recurrence rates of breast cancer within 2 years following chemotherapy cessation, the rationale behind keeping the PORTs in place is the anticipation of potential recurrence necessitating chemotherapy again and a second PORT implantation. On the other hand, to maximize the functionality of PORTs, it is generally recommended to consider PORT removal when the patient’s condition is stable and the risk of recurrence is low [14].

Therefore, this study aimed to explore the timing of PORT removal and the impact of PORT removal within 2 years on patients with breast cancer and PORTs.

## Methods

### Study design and participants

This retrospective study collected breast cancer patients who underwent PORT implantation at our Hospital between July 2012 and May 2022.

The inclusion criteria were 1) 18–80 years of age, 2) histologically confirmed breast cancer treated with systemic chemotherapy, 3) no history of anticoagulant medication, 4) no history of the puncture site or systemic infections, 5) platelet count (PLT) ≥ 50 × 10^9^/L, 6) hemoglobin (HB) ≥ 80 g/L, 7) prothrombin time (PT) < 4 s, 8) Eastern Cooperative Oncology Group (ECOG) performance status (PS) 0–1, who were generally in good condition, and 9) a clear PORT removal timeline. The exclusion criteria were 1) diagnosed or suspected surgical site infection, sepsis, or bacteremia, 2) significant dysfunction of major organs such as heart, lungs, liver, or kidneys making surgery intolerable, 3) coagulation disorders, 4) superior vena cava obstruction syndrome, 5) previous radiation therapy at the planned puncture site, 6) anticipated survival < 3 months, 7) bilateral breast cancer, 8) contralateral recurrence of breast cancer, or 9) incomplete follow-up data.

This study was approved by the Ethics Committee of Zhejiang Cancer Hospital (approval #IRB-2018-149). The requirement for individual consent was waived by the ethics committee due to the retrospective nature of the study.

### Implantation and removal of PORTs

All patients were implanted with 8,806,060 pressure-resistant infusion port central venous catheters (Bard Peripheral Vascular, Inc., Tempe, AZ, USA). The implantation procedure was performed by trained interventional radiologists in the digital subtraction angiography (DSA) operating room. Lymphedema is a possible complication of breast cancer and axillary surgery. Ipsilateral port placement can exacerbate the risk of lymphedema in patients who underwent axillary lymph node dissection due to compromised lymphatic pathways [15, 16]. Therefore, it is common practice at the authors’ center to always place the PORT contralateral to the breast cancer (e.g., on the left for right breast cancer); for bilateral cancers, the PORT was placed in a convenient location that would not interfere with other treatments. The procedure involved ultrasound-guided real-time puncture or direct puncture of the target vessel following ultrasound-assisted localization, routine disinfection, sterile draping, and local anesthesia with lidocaine. Upon successful puncture, a guidewire was inserted through the puncture needle, the catheter was exchanged for the PORTs, and the port distal end was placed between the mid-lower one-third of the superior vena cava and the entrance of the right atrium. A 3-cm incision was made below the puncture site, and a subcutaneous tunnel and pocket were created. The catheter was threaded through the tunnel into the pocket and properly connected to the PORTs before embedding it. After verifying the catheter’s position through contrast imaging and confirming unobstructed infusion with a lossless needle, heparin saline was used to seal the catheter. The wound was disinfected, layered sutures were applied, and the surgery was concluded. The PORTs could be used for intravenous infusion on the day of or the day after the procedure.

The PORTs were flushed with saline to ensure blood return and unobstructed saline injection before use. After infusion, the PORTs were flushed under positive pressure. During treatment intervals, a monthly heparin saline flush was required. PORT removal was conducted under local anesthesia. The surgeon reopened the original incision, separated the subcutaneous tissue, exposed the PORTs, and carefully removed them. After catheter removal, attention was given to checking for PORT breakage or thrombus formation within the PORT wall.

### Data collection and outcomes

Patient data collected included age, breast cancer stage, underlying diseases, body mass index (BMI), puncture pathway, PORT placement site, and PORT implantation and removal dates. Breast cancer staging according to the National Comprehensive Cancer Network (NCCN) guidelines [3]. BMI was calculated as weight divided by height squared. Routine follow-up data were collected, including quality of life (QoL) scores, PORT indwelling time, relevant complications, and medical expenses. QoL scores were assessed using the Karnofsky method [17], categorizing the impact of PORT placement on patients’ lives as follows: Grade A (80–100 points), mostly normal daily life activities; Grade B (60–79 points), mild impact on daily activities; Grade C (< 60 points), moderate to severe impact on daily activities. PORT indwelling time refers to the interval between PORT implantation and removal. Unplanned PORT removal due to complications was termed “unplanned removal,” while PORT removal after completion of intravenous therapy was defined as “planned removal.” Complications included catheter displacement, thrombosis, pinch-off syndrome, infection, poor infusion flow, catheter rupture, incision non-healing, catheter fracture, pain, and so on. Among the complications mentioned above, the infection site could be in a catheter, pocket, or incision infection with a positive bacterial culture. Clinicians and nurses can identify poor infusion flow, non-healing incisions, and pain based on the patient’s symptoms. Pinch-off syndrome is caused by the narrow space between the 1 st rib and the clavicle when the catheter is inserted through the subclavian vein puncture, and the narrowing or clamping caused by the extrusion of the 1 st rib and the clavicle affects the infusion. Catheter rupture, displacement, and fracture can be detected by X-ray. Ultrasound can find thrombosis. Some patients underwent secondary deep vein catheterization after PORT removal for chemotherapy, and the time for this procedure was recorded as the “second catheterization time.” Patients lost to follow-up were censored at the last follow-up date. Follow-up data was collected until March 1, 2023. Medical expenses excluded transportation and diagnostic costs, focusing on total expenditure related to PORT implantation, maintenance, complication management, and removal.

The primary outcome was QoL. The secondary outcomes included the proportion of unplanned PORT removal, secondary venous catheterization, medical costs, and complications.

In breast cancer chemotherapy, after 6–12 months of intensive treatment, maintenance treatment of more than 1 year is often required, for a total of 1.5-2 years [18, 19]. In the endocrine therapy for breast cancer, the SOFT/TEXT trial [20] and ASTRRA trial [21] have shown that even for low-risk patients, endocrine therapy should be maintained for at least 2 years. Therefore, most breast cancer patients need about 2 years of drug treatment, during which they may often need intravenous infusions. Therefore, 2 years is a critical period for breast cancer patients to have an infusion port. Hence, the cut-off value was set at 2 years since in clinical practice, physicians hope that the infusion port can be retained for more than 2 years.

### Statistical analysis

The data were analyzed using SPSS 26.0 (IBM Corp., Armonk, NY, USA). The continuous data were tested for normal distribution using the Kolmogorov-Smirnov test. Continuous data with a normal distribution were expressed as means ± standard deviations (SD) and analyzed using Student’s t-test. Continuous data with a non-normal distribution were expressed as medians (ranges) and analyzed using the Mann-Whitney U-test. Categorical data were presented as *n* (%) and analyzed using the chi-square test. A subgroup analysis was performed among patients with planned and unplanned PORT removal; in each subgroup (i.e., unplanned and planned removal), the patients were divided into two groups: indwelling for ≤ 2 or > 2 years. A Cox multivariable logistic regression analysis was performed to determine the factors independently associated with PORT indwelling time. Two-sided *P*-values < 0.05 were considered statistically significant.

## Results

This study included 376 female patients with a median age of 52 (range, 22–72) years. The median PORT indwelling time was 464 (range, 16-2717) days, and the PORT indwelling time was ≤ 2 years in 252 patients. Ninety patients (23.9%) had stage I breast cancer, 175 (46.5%) had stage II, 69 (18.4%) had stage III, and 42 (11.2%) had stage IV. Ninety-one patients had comorbidities. The BMI was normal in 232 patients, while 144 were underweight or overweight. The median BMI was 23.6 (range, 15.6–33.2) kg/m^2^. Regarding the PORT pathway, 353 patients underwent subclavian vein puncture (196 on the right and 157 on the left), and 23 patients underwent internal jugular vein puncture (21 on the right and two on the left). Regarding the location of PORT placement, 217 were placed on the right chest wall and 159 on the left (Table 1). The reasons for additional secondary catheterization (*n* = 113) were that the patients requested catheterization themselves to facilitate future intravenous medication (*n* = 6), the patients were treated for complications (*n* = 13), and were for anti-tumor treatment (*n* = 94), but the specific data on recurrence, metastasis, or second tumor were incomplete and could not be determined.

**Table 1:** Characteristics of the patients

	*n* = 376
Age	Median: 52 (range, 22 to 72) years Mean: 52.35 ± 9.87 years.
Breast cancer stage*	
Stage I	90 (23.94)
Stage II	175 (46.54)
Stage III	69 (18.35)
Stage IV	42 (11.17)
Comorbidities	
Yes	91 (24.20)
No	285 (75.80)
Body mass index	Median: 23.6 (range, 15.6 to 33.2) kg/m^2^ Mean: 23.61 ± 3.19 kg/m^2^
Normal	232 (61.70)
Low or high	144 (38.30)
Puncture pathway	
Subclavian vein	353 (93.88)
Internal jugular vein	23 (6.12)
Venous port placement site	
Right chest wall	217 (57.71)
Left chest wall	159 (42.29)

Unless stated otherwise in the table, all data are categorical and are shown as *n* (%)

*Clinical staging according to [19]

All patients successfully underwent both PORT insertion and removal, resulting in a 100% success rate for both procedures. The insertion surgery duration ranged from 17 to 75 min, with a median duration of 25 min. The removal surgery duration ranged from 8 to 30 min, with a median of 15 min.

There were no significant differences between the patients with PORT indwelling time ≤ 2 vs. >2 years in terms of age, BMI, comorbidity status, tumor clinical stage, PORT pathway, or PORT catheter location (Table 2) (all *P* > 0.05).

**Table 2:** Comparison of clinical characteristics of patients with PORT indwelling ≤ 2 years and > 2 years

Clinical data	PORT indwelling ≤2 years (*n*=252)	PORT indwelling >2 years (*n*=124)	Odds ratios	*P*
	*n*, %	*n*, %		
Age				
<60	191 (50.80)	98 (26.06)		0.484
≥60	61 (16.22)	26 (6.91)		
Body mass index				
Normal	162 (43.09)	70 (18.62)		0.142
Low or high	90 (23.94)	54 (14.36)		
Clinical staging*				
Ⅰ/Ⅱ	178 (47.34)	87 (23.14)		0.925
Ⅲ/Ⅳ	74 (19.68)	37 (9.84)		
Puncture path				
Subclavian vein	240 (63.83)	113 (30.05)		0.118
Jugular vein	12 (3.19)	11 (2.93)		
Location of port				
Right chest wall	146 (38.83)	71 (18.88)		0.900
Left chest wall	106 (28.19)	53 (14.10)		

All data are categorical and are shown as *n* (%)

*Clinical staging according to [19]

Compared with the patients without complications, higher proportions of age *≥* 60 years (31.87% vs. 20.35%, odds ratio = 1.83, *P* = 0.023), clinical staging III/IV (39.56% vs. 26.32%, odds ratio = 1.83, *P* = 0.016), PORT indwelling *≤* 2 years (78.02% vs. 63.51%, odds ratio = 2.04, *P* = 0.010), and second catheterization (49.45% vs. 23.86%, odds ratio = 3.12, *P* < 0.001) were observed in the patients with complications (Table 3). When considering the patients with stage III or IV disease separately, there were no significant differences in the indwelling time (*≤* 2 years stage III, *n* = 44, 59.5%; *≤*2 years stage IV, *n* = 27, 73.0%; >2 years stage III, *n* = 30, 40.5%; >2 years stage IV, *n* = 10, 27.0%; *P* = 0.162).

**Table 3:** Comparison of clinical characteristics of patients with complication and without

Characteristics	Complications (*n* = 91)	Non complications (*n* = 285)	Odds ratios	*P*
Age				0.023
< 60	62 (68.13)	227 (79.65)		
≥ 60	29 (31.87)	58 (20.35)		
Body mass index				0.480
Normal	59 (64.84)	173 (60.70)		
Low or high	32 (35.16)	112 (39.30)		
Clinical staging*				0.016
Ⅰ/Ⅱ	55 (60.44)	210 (73.68)		
Ⅲ/Ⅳ	36 (39.56)	75 (26.32)		
Puncture path				0.221
Subclavian vein	83 (91.21)	270 (94.74)		
Jugular vein	8 (8.79)	15 (5.26)		
Location of port				0.275
Right chest wall	57 (62.64)	160 (56.14)		
Left chest wall	34 (37.36)	125 (43.86)		
PORT indwelling time				0.010
≤ 2 years	71 (78.02)	181 (63.51)		
> 2 years	20 (21.98)	104 (36.49)		
Second catheterization				< 0.001
No	46 (50.55)	217 (76.14)		
Yes	45 (49.45)	68 (23.86)		

All data are categorical and are shown as *n* (%)

*Clinical staging according to [19]

No significant differences were observed in the QoL scores between patients with PORT indwelling time ≤ 2 vs. >2 years [A/B/C: 90.87%/7.94%/1.19% vs. A/B/C: 95.97%/4.03%/0, *P* = 0.185]. Patients with PORT indwelling time ≤ 2 years exhibited significantly higher probabilities of Unplanned PORT removal (16.76% vs. 3.99%, odds ratio = 2.42, *P* = 0.004), higher secondary venous catheterization (28.99% vs. 3.72%, odds ratio = 5.08, *P* = 0.004), and higher medical costs (16.41 vs. 6.94 CNY/day, *P* < 0.001) compared to those with patients with PORT indwelling time > 2 years. There were no significant differences in the frequency of complications between patients with PORT indwelling time ≤ 2 vs. >2 years (*P* = 0.751) (Table 4).

**Table 4:** Outcomes

	≤ 2 years (*n* = 252)	> 2 years (*n* = 124)	*p*
Quality of life			0.185
A	229 (90.87%)	119 (95.97%)	
B	20 (7.94%)	5 (4.03%)	
C	3 (1.19%)	0	
Reason for removing port			0.004
Planned	189 (75.00%)	109 (87.90%)	
Unplanned	63 (25.00%)	15 (12.10%)	
Second catheterization			< 0.001
No	153 (60.71%)	110 (88.71%)	
Yes	99 (29.29%)	14 (11.29%)	
Median daily fee per patient, CNY	16.41	6.94	< 0.001
Planned port removal (*n*)	189	109	
Median daily fee per patient, CNY	14.83	6.88	< 0.001
Port removal for complication (n)	63	15	
Median daily fee per patient, CNY	24.35	7.72	< 0.001
Complications			0.751
Catheter displacement	13	9	
Thrombosis	15	6	
Pinch-off syndrome	13	3	
Infection	12	2	
Poor infusion flow	7	3	
Catheter rupture	3	0	
Poor wound healing at the insertion site	2	0	
Catheter fracture	1	0	
Pain	1	0	
Tumor invasion of the infusion port	1	0	

Categorical are shown as *n* (%). Non-normally distributed continuous data are shown as median

Subgroup analysis among patients with planned PORT removal revealed that the medical costs for patients with PORT indwelling time ≤ 2 years were significantly higher than in those with PORT indwelling time > 2 years (14.83 vs. 6.88 CNY/day, *P* < 0.001). Similar cost results were observed among unplanned PORT removal cases (Table 4). In addition, after excluding all patients with unplanned extubation, those who underwent PORT removal within 2 years had a significantly higher rate of second catheterization (31.22% vs. 8.26%, *p* < 0.001) and a higher median daily fee per patient (14.83 vs. 6.83 CNY, *p* < 0.001) compared to those with removal after 2 years (Supplementary Table S1).

Among the patients with complications, compared with PORT indwelling > 2 years, those with PORT indwelling *≤* 2 years showed higher proportions of clinical staging III/IV (45.07% vs. 20.00%, odds ratio = 3.28, *P* = 0.043) and second catheterization (56.34% vs. 25.00%, odds ratio = 3.87, *P* = 0.013) (Table 5).

**Table 5:** Comparison of clinical characteristics of patients with complication

Characteristics	PORT indwelling *≤* 2 years (*n* = 71)	PORT indwelling > 2 years (*n* = 20)	*P*
Age			0.197
< 60	46 (64.79)	16 (80.00)	
≥ 60	25 (35.21)	4 (20.00)	
Body mass index			0.116
Normal	49 (69.01)	10 (50.00)	
Low or high	22 (30.99)	10 (50.00)	
Clinical staging			0.043
Ⅰ/Ⅱ	39 (54.93)	16 (80.00)	
Ⅲ/Ⅳ	32 (45.07)	4 (20.00)	
Puncture path			0.999
Subclavian vein	65 (91.55)	18 (90.00)	
Jugular vein	6 (8.45)	2 (10.00)	
Location of port			0.186
Right chest wall	47 (66.20)	10 (50.00)	
Left chest wall	24 (33.80)	10 (50.00)	
Second catheterization			0.013
No	31 (43.66)	15 (75.00)	
Yes	40 (56.34)	5 (25.00)	

All data are categorical and are shown as *n* (%)

The 376 breast cancer patients had a PORT for a total of 260,957 days (average 694 days/case), of which the shortest time was 16 days, the longest was 2717 days, and the median time was 464 days. Among all patients, the average time of wearing the port was 1242.67 ± 65.28 days for those who planned to remove the infusion PORT due to the end of treatment (*n* = 298), and the average time of having the PORT was 625.24 ± 94.35 days for those who did not plan to remove the infusion port due to complications (*n* = 78), with a statistically significant difference (*p* < 0.001). Among the patients with comorbidities such as hypertension, heart disease, and diabetes (*n* = 91), the average time with a PORT was 913.43 ± 110.85 days, and the average time of wearing the port was 1177.20 ± 65.77 days for those without comorbidities (*n* = 285), with statistically significant differences (*p* = 0.017). The PORT time of patients seemed to be only a factor related to the above two factors, but was not significantly correlated with age, body mass index (BMI), clinical stage of tumor, puncture path, and location of port (all *p* > 0.05). A Cox regression analysis showed that complications leading to unplanned port removal were an independent risk factor affecting PORT indwelling time (RR: 2.18, 95%CI: 1.62–2.93, *p* < 0.001).

## Discussion

The optimal timing of PORT removal in cancer patients is unknown. The results suggest that patients with PORT indwelling time ≤ 2 years exhibited significantly higher secondary venous catheterization and higher medical costs compared to those with PORT indwelling time > 2 years. Complications were more common in older patients, in those with more advanced disease, in patients with PORT indwelling time ≤ 2 years, and in those with second catheterization. Moreover, complications that occurred within 2 years were more likely to lead to PORT removal. In those with complications, PORT indwelling *≤* 2 years was associated with older age, more advanced disease, and second catheterization. Therefore, it might be recommended to consider removing the PORTs after 2 years of implantation. The Cox regression analysis showed that complications leading to unplanned port removal were an independent risk factor affecting PORT indwelling time, while age was not independently associated with the indwelling time. Nevertheless, age may play a role in unplanned removal, but additional studies will be necessary.

In accordance with the American Society of Anesthesiologists 2020 Practice Guidelines for Central Venous Access, catheters should be promptly removed when they are no longer clinically necessary or when there is suspicion of catheter insertion site infection [22]. Furthermore, ESMO’s Clinical Practice Guidelines for Central Venous Access in Oncology also mention that catheter removal should be considered in cases of central venous infection, catheter tip migration, or breakage [23]. Combining these viewpoints, PORT removal should be contemplated in two scenarios. Firstly, in the event of severe complications that render the continued use of the PORTs unsuitable. Secondly, even in the absence of complications, the clinical demand for PORTs is no longer sustained. The first criterion is relatively understandable yet lacks specificity regarding the degree of severity of complications that warrant discontinuation of PORT use. It is evident that when a PORT fracture or breakage is detected, it is unnecessary to consider retaining the PORTs. Nevertheless, when complications occur, a choice must be made whether to retain or remove the PORT. The present study observed that unplanned removal due to complications was associated with the duration of PORT retention. The most common PORT complications are infection, thrombosis, and catheter displacement [24, 25]. Infection and thrombosis are frequent complications of any central venous catheter [26, 27]. Catheter-related thrombosis generally does not lead to fatal pulmonary embolism but may render the catheter nonfunctional. In most cases, thrombus dissolution within the catheter can be achieved through repeated urokinase flushing, thus enabling continued infusion. Still, when the thrombus burden is substantial or recurrent thrombosis occurs, the benefits of catheter removal become more pronounced. Infections localized to the pocket site can be managed through wound cleaning and dressing changes. On the other hand, catheter-related bloodstream infections represent more severe complications, often warranting catheter removal as the preferred course of action. Notably, there was an almost insignificant difference in the frequency of complications like infection and thrombosis between jugular and subclavian vein punctures [28–30], supporting the present study.

Catheter displacement often leads to impaired infusion and escalates the risk of catheter damage and thrombosis. Therefore, every effort should be made to reposition the catheter. Techniques involve using pigtail, cobra, or gooseneck catheters to hook onto the displaced catheter and pull it back to its original position, with a success rate exceeding 90% [31]. Nevertheless, catheter removal can be considered if catheter repositioning proves challenging or recurrent displacements occur. As previously mentioned, most complications do not lead to catheter dysfunction. If local interventions successfully maintain catheter function, there is no immediate need for PORT removal. Through drug thrombolysis, anti-infection measures, and interventional techniques, over half of the cases involving complications such as infection, thrombosis, and catheter displacement were successfully managed in the present study. Nevertheless, since this study exclusively focused on patients who already had their PORTs removed, and since many patients who could keep their PORTs were not included in the study, the frequency rate of complications might be overestimated. In fact, complications leading to PORT removal remain relatively rare. One point worth making clear is that the PORT removal within 2 years reduces the occurrence of complications, but the type of complications occurring within 2 years is more likely to lead to PORT removal.

Older age, more advanced breast cancer, PORT indwelling *≤* 2 years, and a second catheterization were associated with PORT complications. A study in patients with gastric cancer showed that worse performance status, high albumin levels, no implantation procedure, and implantation not performed by specialized groups were risk factors for PORT complications [32]. No therapeutic anticoagulation, PORTs on the left side, and insertion as an inpatient have been associated with higher risks of PORT complications [33]. Yu et al. [34] reported that age and breast, lung, and gastric cancers were risk factors for late PORT complications. Of course, the differences in the variables collected in the studies prevent any direct comparisons.

The second guideline criterion suggests that when intravenous access is no longer clinically necessary, the consideration of PORT removal arises, but the exact optimal timing remains unknown. Based on the results of the present study, a timeline of 2 years appears reasonable. Indeed, this study revealed that among those who underwent planned PORT removal within 2 years, the rate of subsequent PORT re-implantation reached 34.39%. In contrast, among those who had their PORTs removed after 2 years, the rate of subsequent re-implantation was notably lower at 9.17%. This difference between the two groups was statistically significant. In reality, the procedure of PORT removal is straightforward, carries a high degree of safety [35], and is often conducted in an outpatient surgical setting [36]. In the absence of active bleeding, many patients even bypass the need for preoperative blood tests [37]. Consequently, PORT removal is relatively convenient and cost-effective. Nevertheless, the occurrence of re-implantation within a short period not only increases patient discomfort but also significantly escalates medical expenses. For patients with breast cancer who frequently rely on deep venous access, it is advisable not to rush PORT removal within the first 2 years after treatment completion. Instead, considering removal beyond 2 years when intravenous therapy is no longer necessary may be a more appropriate course of action.

Health economics studies demonstrated that beyond a retention period of 9–12 months, PORTs prove more cost-effective than PICCs [38]. Hence, the removal of PORTs within 1 year lacks financial prudence. In alignment with the present study, patients undergoing planned PORT removal within 2 years exhibited significantly higher daily medical expenses associated with the PORTs compared to those whose PORT removal was planned after 2 years, and the rate of secondary catheterization was also higher in those with *≤* 2 years of PORT indwelling. Still, there were no significant differences in quality of life or complications. Nevertheless, PORT removal within 2 years seems economically suboptimal, with greater advantages in medical costs incurred by planned removal after this timeframe. The results suggest that even when excluding the factors related to unplanned extubation, patients with an indwelling time of > 2 years may still benefit from PORT implantation.

This study had some limitations. The patients were from a single institution, leading to a relatively small sample size when considering the high incidence of breast cancer and the use of PORTs. Only patients with breast cancer were included. Patients with breast cancer have a long survival compared with other cancers [1, 2], limiting the generalizability of the results to cancer patients. In the present study, some patients received a PORT as early as 2012, but treatment recommendations based on the molecular classification (e.g., luminal A, basal-like, etc.) were formulated only in 2016 in China, preventing subgroup analyses. The study was based on the local patient charts, and whether complications occurred and were managed at another hospital cannot be excluded. In a similar manner, the adherence to the monthly flushing is unknown since it could be done at a local hospital. Subgroup analyses were performed, but because of the relatively small sample size, stage III and IV cancers were grouped together despite the different intents of systemic therapy (curative vs. palliative) and the fact that patients with metastatic disease are more likely to receive ongoing therapy. Patients with de novo stage IV disease are less likely to have undergone axillary procedures that could lead to issues with vascular access.

## Conclusion

In patients with breast cancer who do not require regular intravenous administration, it may be recommended to consider removing the infusion port after 2 years to reduce the risks of secondary catheterization and reduce medical costs, unless irreversible adverse events affect the correct use of the PORT. A multicenter prospective study with large samples was needed to verify the results.

## Supplementary Information

Supplementary Material 1.

## Data Availability

All data generated or analyzed during this study are included in this article.

## Abbreviations

PORTs: Optimal timing of implanted vascular access ports
QoL: Quality of life
TIVAD: Totally implantable venous access devices
HB: Hemoglobin
ECOG: Eastern Cooperative Oncology Group
PS: Performance status
DSA: Digital subtraction angiography
BMI: Body mass index
NCCN: National Comprehensive Cancer Network
SD: Standard deviations
