# Supplementary material for: When to remove implantable vascular access ports? a retrospective analysis of 376 patients with breast cancer and implantable vascular access ports
Source: BMC Surg. 2025 Oct 3;25:420. doi: 10.1186/s12893-025-03173-4 (PMC12495737; doi:10.1186/s12893-025-03173-4)
Supplement: Supplementary file 1 — Supplementary Material 1. [file 12893_2025_3173_MOESM1_ESM.docx]

**Supplementary Table S1.** Outcomes among patients with planned PORT removal

|  | ≤2 years (n=189) | >2 years (n=109) | p |
| --- | --- | --- | --- |
| Quality of life |  |  | 0.495 |
| A | 187 (98.94%) | 109 (100%) |  |
| B | 2 (1.06%) | 0 |  |
| C | 0 | 0 |  |
| Second catheterization |  |  | <0.001 |
| No | 130 (68.78%) | 100 (91.74%) |  |
| Yes | 59 (31.22%) | 9 (8.26%) |  |
| Median daily fee per patient, CNY | 14.83 | 6.83 | <0.001 |
| Complications |  |  | 0.070 |
| Catheter displacement | 1 | 0 |  |
| Thrombosis | 0 | 3 |  |
| Pinch-off syndrome | 7 | 2 |  |

Categorical are shown as n (%). Non-normally distributed continuous data are shown as median.
